# Specific Antibody Production by Blood B Cells is Retained in Late Stage Drug-naïve HIV-infected Africans

**DOI:** 10.1080/10446670410001722104

**Published:** 2004-06

**Authors:** Lydie Béniguel, Evelyne Bégaud, Fabrice Cognasse, Philippe Gabrié, Christophe D. Mbolidi, Mary A. Marovich, Céline Cazorla, Frédéric Lucht, Christian Genin, Olivier Garraud

**Affiliations:** 1 GIMAP EA3064 Faculté de Médecine Université J. Monnet St-Etienne France; 2 Institut Pasteur de Bangui Bangui Central African Republic; 3 Hôpital Communautaire Bangui Central African Republic; 4 Combined US Military HIV Research Program Rockville MD USA; 5 Service des Maladies Infectieuses CHU. St-Etienne France

## Abstract

Unseparated peripheral blood mononuclear cells (PBMCs) obtained from drug-naïve African individuals living in a context of multi-infections and presenting with high viral load (VL), were cultured *in vitro* and tested for their ability to produce antibodies (Abs) reacting with HIV-1 antigens. Within these PBMCs, circulating B cells were differentiated *in vitro* and produced IgG Abs against not only ENV, but also GAG and POL proteins. Under similar experimental conditions, HAART treated patients produced Abs to ENV proteins only. The *in vitro* antibody production by drug-naïve individuals' PBMCs depended on exogenous cytokines (IL-2 and IL-10) but neither on the re-stimulation of reactive cells in cultures by purified HIV-1-gp 160 antigen nor on the re-engagement of CD40 surface molecules. Further, it was not abrogated by the addition of various monoclonal Abs (mAbs) to co-stimulatory molecules. This suggests that the *in vitro* antibody production by drug-naïve individuals' PBMCs resulted from the maturation of already envelope and core antigen-primed, differentiated B cells, presumably pre-plasma cells, which are not known to circulate at homeostasy. As *in vitro* produced Abs retained the capacity of binding antigen and forming complexes, this study provides pre-clinical support for functional humoral responses despite major HIV- and other tropical pathogen-induced B cell perturbations.


*Clinical & Developmental Immunology*, June 2004, Vol. 11 (2), pp. 121–127

Taylor & Francis healthsciences

### Specific Antibody Production by Blood B Cells is Retained in Late Stage Drug-naïve HIV-infected Africans

LYDIE BÉNIGUEL[a], EVELYNE BÉGAUD[b], FABRICE COGNASSE[a], PHILIPPE GABRIÉ[c], CHRISTOPHE D. MBOLIDI[c], MARY A. MAROVICH[d], CÉLINE CAZORLA[e], FRÉDÉRIC LUCHT[e], CHRISTIAN GENIN[a] and OLIVIER GARRAUD[a,*]

[a]*GIMAP EA3064, Faculté de Médecine, Université J. Monnet, St-Etienne, France;* [b]*Institut Pasteur de Bangui, Bangui, Central African Republic;* [c]*Hôpital Communautaire, Bangui, Central African Republic;* [d]*Combined US Military HIV Research Program, Rockville, MD, USA;* [e]*Service des Maladies Infectieuses, CHU, St-Etienne, France*

Unseparated peripheral blood mononuclear cells (PBMCs) obtained from drug-naïve African individuals living in a context of multi-infections and presenting with high viral load (VL), were cultured *in vitro* and tested for their ability to produce antibodies (Abs) reacting with HIV-1 antigens. Within these PBMCs, circulating B cells were differentiated *in vitro* and produced IgG Abs against not only ENV, but also GAG and POL proteins. Under similar experimental conditions, HAART treated patients produced Abs to ENV proteins only. The *in vitro* antibody production by drug-naïve individuals' PBMCs depended on exogenous cytokines (IL-2 and IL-10) but neither on the re-stimulation of reactive cells in cultures by purified HIV-1-gp160 antigen nor on the re-engagement of CD40 surface molecules. Further, it was not abrogated by the addition of various monoclonal Abs (mAbs) to co-stimulatory molecules. This suggests that the *in vitro* antibody production by drug-naïve individuals' PBMCs resulted from the maturation of already envelope and core antigen-primed, differentiated B cells, presumably pre-plasma cells, which are not known to circulate at homeostasy. As *in vitro* produced Abs retained the capacity of binding antigen and forming complexes, this study provides pre-clinical support for functional humoral responses despite major HIV- and other tropical pathogen-induced B cell perturbations.

*Keywords*: Anti-HIV Antibody; B cell; IVAP; PBMC

*Abbreviations*: CG B cells, centro germinative B cells; IVAP, *in vitro* antibody production; WB, Western blot; VL, viral load

### INTRODUCTION

HIV-infection in Africa presents some unique features including diverse viral clades and clinical manifestations of disease. The limited access to HAART and also the presence of chronic tropical diseases induce profound lymph node and germinal center disorganization. An important issue in order to take care of these patients is to determine if they are still capable of producing functional HIV-specific antibodies (Abs), and—at large—functional Abs to other pathogens.

HIV infection induces disturbances in cellular and humoral immunity, both at the specific and non-specific levels (Fauci *et al.*, 1996). Typically, untreated HIV-infected individuals experience disease progression accompanied by hypergammaglobulinemia yet lack specific neutralizing anti-HIV envelope glycoprotein Abs. It is commonly held that a profound B cell dysfunction is associated with HIV (Richard *et al.*, 2002). Meanwhile, there is an increasing body of evidence that certain neutralizing Abs (NAbs) are instrumental in protection against HIV infection, as demonstrated both at the mucosal and the systemic level (Devito *et al.*, 2000; Moja *et al.*, 2000). NAbs may indeed achieve protection in the systemic compartment, at least in certain experimental hosts (Mascola *et al.*, 1999; Baba *et al.*, 2000).

We have previously shown that HIV-infected, drug-naïve AIDS patients living in Africa, and exposed to multiple concurrent infections (Garin *et al.*, 1997; Germani *et al.*, 1998; Begaud *et al.*, 2003), exhibited diminished levels of naïve and memory-blood B lymphocytes but augmented levels of centro-germinative (CG), post-germinative blood B cells, and pre-plasma cells which are aberrant cells in the periphery (Beniguel *et al.*, 2004).

The present investigation aimed at examining whether peripheral blood mononuclear cells (PBMCs) obtained

*Corresponding author. Address: GIMAP, Faculté de Médecine, 15 Rue A. Paré, 42023 St-Etienne Cédex 02, France. Tel.: +33-04-77-42-14-67. Fax: +33-04-42-14-86. E-mail: olivier.garraud@univ-st-etienne.fr

ISSN 1740-2522 print/ISSN 1740-2530 online © 2004 Taylor & Francis Ltd
DOI: 10.1080/10446670410001722104

from drug-naïve HIV-infected Africans presenting with AIDS and comprising of various B cells subsets—of which usually non-circulating B cells—could still produce HIV-1-specific Abs. The study also aimed at examining some characteristics of this *in vitro* Ab production (IVAP) to set up a rationale for immune-interventions when available.

### MATERIALS AND METHODS

### Patients

African donors were HIV-positive patients from the "Hôpital Communautaire" of Bangui, Central African Republic (CAR). The prevalence of HIV-infection in CAR is estimated to be 12.9% of the adult population (http://www.unaids.org; 2002). All donors enrolled in this study were informed and consented to donate blood for research purposes, according to the rules of the *ad hoc* Ethics Committee set up by the Institut Pasteur in Bangui, in the absence of a National Ethics Committee. None of the blood donors were previously diagnosed with HIV infection, and all were indeed antiretroviral drug-naïve.

For comparison of biological parameters, European volunteer blood donors were also tested in this survey. They consisted of 10 HIV-positive patients (Table I) followed by the Department of Infectious Diseases at the University Hospital in St-Etienne. Informed consent was obtained from every patient according to the requirements of the National French Ethics Committee. They were all treated with antiretroviral drugs according to current protocols. Each European patient was also monitored for clinical and biological parameters predictive of disease progression. Matched controls consisted of HIV-negative African and European blood donors. Approximately 10 ml of heparinized blood was obtained for the present study.

TABLE I Major characteristics of the $HIV^{+}$ patients enrolled in the study. (A) African patients, (B) European patients

| | | Age | CD4 | VL |
|---|---|---|---|---|
| (A) African patients | | | | |
| 1 | F | 30 | nd | 5.15 |
| 2 | F | 28 | 246 | 5.88 |
| 3 | F | 18 | 1117 | nd |
| 4 | M | 45 | 133 | 5.51 |
| 5 | F | 43 | nd | nd |
| 6 | M | 33 | 65 | nd |
| 7 | F | 27 | 421 | nd |
| 8 | M | 33 | 23 | 5.88 |
| 9 | F | 30 | 445 | 5.4 |
| 10 | F | 22 | nd | 5.61 |
| 11 | M | 56 | nd | nd |
| 12 | F | 20 | 854 | nd |
| 13 | M | 32 | 378 | nd |
| 14 | F | 24 | 702 | nd |
| 15 | M | 32 | 9 | 4.2 |
| 16 | F | 38 | 268 | 5.2 |
| 17 | F | 16 | 992 | nd |
| 18 | M | 39 | 456 | 5.4 |
| 19 | F | 28 | 128 | nd |
| 20 | M | 33 | 68 | nd |
| 21 | F | 28 | 854 | nd |
| 22 | F | 25 | 956 | 4.5 |
| 23 | M | 43 | 1332 | 5.6 |
| 24 | F | 38 | 611 | 5.15 |
| (B) European patients | | | | |
| 1 | M | 64 | 499 | > 1.7 |
| 2 | M | 44 | 303 | 3.81 |
| 3 | F | 49 | 759 | > 1.7 |
| 4 | M | 39 | 383 | > 1.7 |
| 5 | M | 31 | 650 | 4.52 |
| 6 | M | 46 | 439 | > 1.7 |
| 7 | M | 41 | 415 | 3.63 |
| 8 | F | 66 | 442 | > 1.7 |
| 9 | M | 45 | 322 | > 1.7 |
| 10 | M | 32 | 465 | 3.85 |

Mean $CD4^{+} = 502.9 \pm 403.2$ cells/μl. Mean VL = 5.24. Mean $CD4^{+} = 468 \pm 134$ cells/μl. Mean VL = 2.6.

*Notes*: M and F stands for Male and Female; Age is expressed in years; CD4 count is expressed in $CD4^{+}$ cells per μl ± SD; plasma viral load is expressed in log (copies/ml).

nd: not determined.

### Serology and Viral Load (VL) Determination

HIV serology was routinely followed in Bangui using the Vironoctika HIV Uniform II plus O test (Organon Teknika, Durham, NC). HIV serology in St-Etienne was done with the HIV duo (Vidas Biomérieux, Marcy L'Etoile, Lyon, France) and Enzygnost HIV1 + 2 (Behring, Marburg, Germany). Plasma VL was determined using a PCR technique as previously described (Bourlet *et al.*, 2001).

### PBMC Preparation

Plasma was removed by centrifugation at 1,500 rpm/min and stored at −20°C. PBMCs were isolated by density gradient centrifugation (Lymphocyte Separation Medium, Eurobio, Les Ulis, France). As the B cell phenotyping was not performed in Bangui, $1 \times 10^6$ PBMC were stored at 4°C in Stabilcyte™ (BioErgonomics, St Paul, MN), for approximately 2 months prior to be shipped in France and labeled for flow cytometry analysis.

### PBMC Cultures

PBMCs were cultured in Iscove's Modified Dulbecco's Medium (IMDM) culture medium (Biowhittaker, Verviers, Belgique) supplemented with 10% endotoxin-free heat-inactivated Fetal Calf Serum (FCS) (US14-901F; BioWhittaker), 1% MEM amino acids solution (Sigma-Aldrich, Dorset, UK), 1% MEM non-essential amino acids solution (Sigma), 1% Insulin–Transferin–Selenite medium (Sigma) and 1% antibiotics (Antibiotic–Antimytotic solution; Sigma) as described (Garraud *et al.*, 1995). This preparation will therefore be referred to as "complete medium". In order to allow cultured PBMCs to shed membrane-bound Igs or immune complexes that could interfere with specific assays, PBMC were rested by an overnight incubation at 37°C as already described (Garraud *et al.*, 2001). On the following day, PBMCs were readjusted at $10^6$ cells/ml in fresh cultured medium and 500 μl was added to each well of a 48-well culture plate (Costar, NY).

PBMCs were then cultured in the presence or absence of additional stimuli to favor the terminal differentiation of B cells into Ig secreting cells. The following stimuli were used alone or in combinations at predetermined concentrations: (i) cytokines: IL-4 (Peprotech, Rocky Hill, NJ); IL-2 (Peprotech); IL-10 (R&D Systems, Minneapolis, MN) and TGF-β, all used at 20 ng/ml. (ii) Ag: HIV MN/LAI Ag gp160 (Aventis-Pasteur, Marcy-L'Etoile, France), used at 0.1 μg/ml; (iii) control cultures were performed in the presence of cycloheximide (CHX) (0.1 mg/ml; Sigma) to prevent protein synthesis. Some PBMC were also stimulated with 10 ng/ml of soluble CD40L molecule. Individual cultures were carried out for 3–12 days at 37°C in 5% $CO_2$ humidified atmosphere. Cell-free culture supernatants were stored at −20°C until used in Ab detection assays.

To test for the binding capacity of *in vitro*-produced anti-gp160 IgG Abs, individual 200 μl supernatant samples were incubated with purified recombinant gp160 (used at 100 ng as determined in a series of preliminary experiments), for 3 h at 37°C, prior to Western blotting (WB).

In an attempt to inhibit Ag presentation to B cells and/or direct cell adhesion, PBMCs were cultured in 96-well plates in the presence or absence of anti-CD54, anti-CD18, anti-CD11c and anti-HLA-DR mAbs, or isotype controls (Coulter-Immunotech, Marseille, France). These monoclonal Abs (mAbs) were used at a predermined concentration of 1 μg/ml at the onset of the cultures.

### Apoptosis Assays

In order to estimate ongoing apoptosis of cultured cells, culture supernatants (100 μl) were tested *a posteriori* for the presence of NMP 41/7 (Nuclear Matrix Protein, a protein released by cell death) in culture supernatants, using a commercial NMP ELISA (Calbiochem, San Diego, CA). It has been described that the presence of NMP41/7 marker, in cell culture supernatants, was correlated to cell DNA fragmentation and FAS-Ligand production (Baize *et al.*, 1999).

### Ab Production Analysis

For the broad detection of anti-HIV IgG Abs, plasma (dilution 1/90) or culture supernatants (dilution 1/9) were tested by WB (HIV BLOT 2.2, Abbott, Rungis, France) according to the manufacturer's protocol. Briefly, samples were incubated overnight on specific nitrocellulose blots in the appropriate buffer. Positive and negative controls were high anti-HIV Ab titer serum and HIV-negative serum, respectively. After several washes, blots were incubated 30 min with a phosphatase alkaline conjugated anti-IgG and 15 min with the appropriate substrate.

Additionally, total IgG in culture supernatants were analyzed using ELISAs performed in 96-well MaxiSorp™ plates (NUNC, Roskilde, Denmark) as previously described (Garraud *et al.*, 1995). IgG concentration was extrapolated from a reference curve generated by assaying dilutions of a pool of plasma specimens from blood donors whose IgG concentrations were determined by an immunonephelometric technique (Minineph™, The Binding Site, Oxford, UK).

### PBMC Phenotyping

Standard T ($CD4^+$) cell phenotyping was performed at the Institut Pasteur in Bangui using 100 μl of fresh whole blood labeled with a mAb (anti-CD4, Becton Dickinson Immunocytometry Systems, San Jose, CA) as described (Menard *et al.*, 2003). Lymphocyte subsets were routinely analyzed on a FACSCalibur flow cytometer (Becton Dickinson Immunocytometry Systems).

As B cell phenotyping was not routinely performed in Bangui, PBMC were stored in Stabilcyte™ until the staining. After extensive washings in PBS with 10% FCS, PBMC ($0.5 \times 10^6$) were labeled with RPECy5 labeled anti-CD20 mAb (DAKO, Copenhagen, Denmark). Briefly, $0.5 \times 10^6$ cells were stained with the mAb for 45 min on ice. Afterwards, the cells were washed in PBS/10% FCS and fixed in PBS containing 4% paraformaldehyde. Flow cytometry was performed on the following day using a FacsVantage-SF BD and the CellQuest-Pro™ software (Becton-Dickinson).

### Statistical Analysis

Means ± standard deviations (SD) of total IgG in individual cultures were compared by means of the Wilcoxon test (Statview™, Cary, NJ).

### RESULTS

### Characteristics of the Blood Donors

The mean $CD4^+$ T cell count ± SEM was 502.9 ± 403.2 cells/μl for $HIV^+$ African donors and 913 ± 66 cells/μl for $HIV^-$ African donors. The % mean of $CD20^+$ B cell count ± SEM was 4.9 ± 0.7 for the $HIV^+$ African donors and 6.7 ± 0.6 for $HIV^-$ African donors. As expected, the plasma VL was high for drug-naïve African patients. It ranged from 10,000 to more than 800,000 copies/ml. For HAART-European patients, plasma VL was controlled by antiretrovirals and ranged from less than 50 to 50,000 copies/ml, with 6 patients having less than 50 copies/ml (Table I).

### Ig Production *In Vitro*

PBMCs from $HIV^+$ and $HIV^-$ African donors produced equally large amounts of Ig *in vitro* (mean IgG production were 0.115 mg/l) as tested by ELISA. This IVAP was not statistically augmented by exogenous cytokines IL-2 and IL-10 and/or by recombinant HIV-gp160. As previously shown, the Ig production *in vitro* in HIV$^+$ individuals was detected as early as day 3 whereas maximum production was observed after a 12 day culture (Cognasse *et al.*, 2003). This production was abrogated by CHX, indicating it was *de novo* protein synthesis.

### Specific Ab Production *In Vitro* by Peripheral Blood B Cells from HIV$^+$ Individuals

PBMCs from 14 drug-naive African HIV$^+$ donors (Table I; (1–14) were cultured *in vitro* for 12 days in the absence of or presence of exogenous stimuli, IL-2 + IL-10 or HIV-1-gp160 Ag. In the vast majority (12/14) of individual cultures, there was a spontaneous production of anti-HIV IgG Abs, which was fairly augmented by addition of IL-2 and IL-10 at the onset of the cultures. For the two other individual cultures, a spontaneous Abs production could also be detected by WB but was not augmented by IL-2 and IL-10. In the 14 cultures, no specific anti-gp160 Ab production was detected when HIV-gp160 was added at the onset of the cultures. These data are summarized in Table IIA. Thus the overall profile of IVAP in drug-naïve HIV-infected Africans' PBMC was a spontaneous Ab production which was augmented at least in terms of antigenic specificities of Ab by IL-2 and IL-10 (Fig. 1A). Of important note, soluble CD40L did not prove to augment IVAP under these conditions.

Characteristically, the specific *in vitro* induced Abs could recognize blotted Ags corresponding to gp160, gp41 (ENV-), p24, p17 (GAG-) and p66, p51 and p31 (POL-proteins). This broad distribution profile of Ab specificities was observed in all 14 productive cultures.

In contrast, when PBMCs from HAART-treated patients (Table IB) were cultured, three distinct profiles of IVAP could be observed. In 2/10 individual cultures there was no production of Ab. In the other 8/10 cultures, there was a detectable spontaneous Ab production against ENV Ag. This production could be augmented by IL-2 and IL-10 in 3/8 cases but could not in the other 5/8 cases, 4 of these 5 corresponding to individual cultures of PBMCs from donors with less than 50 mRNA copies/ml of plasma. These data are summarized in Table IIB. The three individual cultures where IVAP was augmented by IL-2 and IL-10 were performed using PBMCs from donors with more than 2,500 mRNA copies/ml of plasma. Of important note, 8/8 productive individual cultures contained Abs which could recognize blotted Ags corresponding to gp160 only, and only 1/8 combined a mixture of anti-p31 (POL-protein) and anti-gp160 (ENV-protein) and only 1/8 combined a mixture of anti-p24 (GAG-protein) and anti-gp160 Abs (Fig. 1).

The kinetics of Ab production was next assayed in a novel series of cultures. This showed that Ab production was detectable in culture supernatants as early as day 3, in contrast to what is generally observed in PBMC cultures from HIV$^+$, but treated, donors.

### Exogenous HIV-gp160 Extinguishes Spontaneous Ab Production in HIV$^+$ Patients

In the majority of the culture systems described so far, addition of a relevant stimulating Ag was required for PBMCs to produce Ag-specific Abs *in vitro* (Garraud *et al.*, 1996). A stated above, "spontaneous" production of Ag-specific Abs was observed in the absence of stimulating Ag (e.g. recombinant gp160). Rather, in the presence of Ag, the Ab production was abolished, an observation which was made in every individual culture (Fig. 1). As Ag overstimulation can induce apoptosis in B cells, we tested *a posteriori* for the presence of NMP41/7 in every culture supernatant. No evidence of apoptosis or at least cell death was detected in any individual culture.

We next tested for the possibility that specific anti-gp120/160 Abs were released in culture supernatants earlier than usual when naïve B cells undergo the complete pathway of cell differentiation *in vitro*. An indirect approach consisted of testing for the capacity of *in vitro*-produced Abs to make immune complexes with Ag. Culture supernatants clearly showing spontaneous Ab production were incubated *a posteriori* with recombinant gp160 for 3 h. This operation led to the complete adsorption of anti-gp120/160 Abs in every culture supernatant tested as can be seen in Fig. 1B.

TABLE II Antigenic specificities of the Ab produced by PBMC *in vitro*

| | IVAP in individual PBMC cultures in drug-naïve Africans | | | | IVAP in individual PBMC cultures in HAART-Europeans | | | |
|---|---|---|---|---|---|---|---|---|
| | Additional stimulus | | | | Additional stimulus | | | |
| Antigen reactivity | CHX | ns | IL-2 IL-10 | HIV-1 gp160 | CHX | ns | IL-2 IL-10 | HIV-1 gp160 |
| gp160 | 0/14 | 14/14 | 14/14 | 0/14 | 0/10 | 8/10 | 8/10 | 0/10 |
| p66 | 0/14 | 1/14 | 7/14 | 1/14 | 0/10 | 0/10 | 0/10 | 0/10 |
| p51 | 0/14 | 1/14 | 5/14 | 1/14 | 0/10 | 0/10 | 0/10 | 0/10 |
| gp41 | 0/14 | 0/14 | 3/14 | 0/14 | 0/10 | 0/10 | 0/10 | 0/10 |
| p31 | 0/14 | 0/14 | 6/14 | 0/14 | 0/10 | 0/10 | 1/10 | 0/10 |
| p24 | 0/14 | 2/14 | 3/14 | 2/14 | 0/10 | 0/10 | 1/10 | 0/10 |
| p17 | 0/14 | 0/14 | 1/14 | 0/14 | 0/10 | 0/10 | 0/10 | 0/10 |

*Note*: The Ab production in three culture conditions was estimated according to WB in the two groups of patients. The number of patients producing the specific Ab are indicated. ns stands for non-stimulated PBMC.

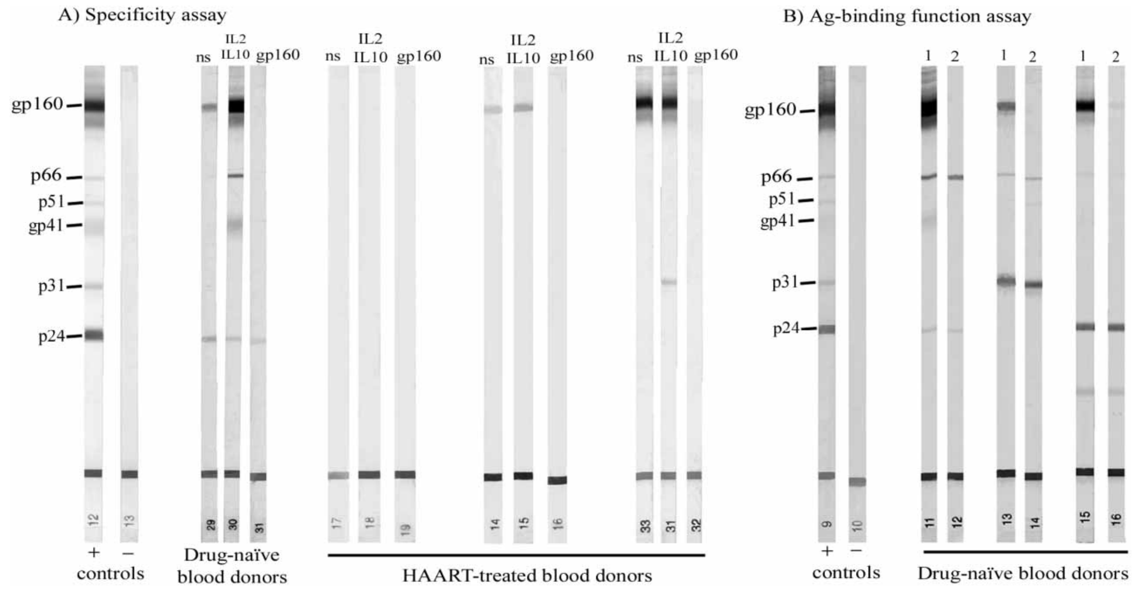

FIGURE 1 (A) *Specificity assay*. This figure shows HIV blots of PBMC culture supernatants for HIV$^+$ drug-naïve and HAART-treated patients in various culture conditions: "ns" (non-stimulated) stands for PBMC in the absence of exogenous stimulus; "IL-2, IL-10" stands for PBMC stimulated by IL-2 and IL-10; "gp160" stands for PBMC cultured in the presence of HIV-1 gp160. C + stands for the positive-control; C − stands for the negative-control. A typical drug-naïve profile are presented; 3 Ab profile for HAART-treated patients are presented. (B) Ag-binding function assay. Supernatants of individual cultures of 3 drug-naïve patients are indicated. (1) Stands for the supernatant of PBMC stimulated by IL-2 and IL-10; (2) represents the same supernatants after a 3 h incubation with an excess of HIV-1 gp160. C + stands for the positive-control; C − stands for the negative-control.

In contrast, anti-GAG and anti-POL proteins remained unchanged.

### Differential Intercellular Cooperation Affecting Spontaneous Specific Ab Production *In Vitro*

The above reported data showed that: (i) HIV$^+$ African individuals' PBMCs produced anti-HIV Ab, against ENV proteins but also against GAG and POL proteins; (ii) such Abs, characterized as IgG, were functional since they bound exogenous gp160; (iii) in the majority of individual cultures, this Ab production was augmented by exogenous IL-2 and IL-10. We next examined the role of intercellular molecular events between lymphocytes and other cell types in PBMCs from HIV$^+$ patients. PBMCs from 10 Africans (Table I, 14–24) were incubated with a cocktail of anti-leukocyte adhesion molecules (i.e. anti-CD54, anti-CD18, anti-CD11c, and anti-HLA-DR), in the presence or absence of IL-2 and IL-10 and in the presence or absence of gp160. The mAb cocktail was added at the onset of the 12-day PBMC culture. Spontaneous anti-ENV, as well as anti-POL and anti-GAG, proteins remained unaffected in every individual culture. Although it cannot be excluded that mAbs were insufficient in amounts, or that repetitive addition of this cocktail over time would have been necessary, or that other intercellular Ag specificities would have been of greater effect, those experiments suggest that spontaneous Ab production *in vitro* by HIV$^+$ individuals' PBMCs is relatively independent of Ag presentation (at least by non B-cells), but is dependent on certain accessory cell-derived soluble factors such as cytokines. In a limited number of experiments, similar results were obtained when monocytes were depleted from PBMCs by adhesion to plastic prior to the culture steps (data not shown).

### DISCUSSION

Early in the AIDS epidemic, one commonly reported biological disturbance was hypergammaglobulinemia. Spontaneous production of Ig of diverse, but often irrelevant, specificities, has been recorded when PBMCs of HIV-infected individuals were examined in short- or longer-term cultures (Amadori *et al.*, 1988; Vendrell *et al.*, 1991). The African environment of concurrent infections may impact humoral responses at large, as shown by total Ig production in HIV$^+$ and HIV$^-$ donors. This is related to observations of immune activation in a tropical environment (Lukwiya *et al.*, 2001).

Observations over time have also been challenged with the considerable progress achieved in managing the patients, such as HAART and consistent reduction of VL (Opravil *et al.*, 2002). The machinery involved in such Ab production dysfunction, however, most probably resides in the LNs.

Our studied population comprised of African blood donors unaware of their HIV$^+$ status although they displayed characteristics of AIDS infection as they were recruited on the basis of the WHO classification for adult AIDS in Africa (Greenberg *et al.*, 1997).

We previously shown—in the same African population—that HIV$^+$ patients displayed an abnormal circulating blood B cell phenotype. Within the circulating

B cell pool there was an aberrant contingent of ($CD20^+/CD77^+$, $sIgM^-$ $sIgG^-$ $sIgA^-$ $sIgD^+$) CG and ($CD20^+/CD38^+$ $CD138^+$) post-CG B cells usually restricted to lymph nodes or equivalent secondary lymphoid organs (Beniguel *et al.*, 2004). In general, at homeostasis, CG B cells are prone to apoptosis unless they (i) encounter specific Ag; (ii) receive a CD40/CD40L signal; (iii) receive activated T cell-mediated signals; and/or (iv) are in contact with follicular dendritic cells which provide Ag stimulation and other cognate and secretory signals (Defrance *et al.*, 2002).

In the present study, we questioned whether untreated $HIV^+$ African patients' peripheral B cells were able to differentiate *in vitro* and produce anti-HIV Abs, as those B cells comprised of numerous CG and post-CG cells which are usually prone to apoptosis, along with naïve and memory B cells (which can be rather easily differentiated *in vitro*). As there was no evidence of cell death in the cultures, it cannot be excluded that most of those cells, which were prone to apoptosis, died earlier on at the time of the 24 h resting period preceding the culture. Cultured cells could produce fair levels of anti-HIV Abs in the presence of survival signals such as IL-2 and IL-10 but did not necessitate the re-engagement of CD40 and the re-exposure to virus-derived Ags. This strongly suggest that at least one major sub-population of B cells prone to differentiate terminally were in fact already differentiated and matured in such a manner that they produced not only anti-ENV but also anti-GAG and POL-Abs. This observation was strengthened by the observation that the terminal maturation during the IVAP process was independent on costimulatory molecules. It is thus very likely that this population of B cells could comprise of pre-plasma cells, whose % was elevated, an observation already made by Fournier *et al.* (2002). Pre-plasma cells do not express CD40, and would thus be unsensitive to exogeneously supplied soluble CD40L signals.

Interestingly, our results indicate that B cell priming of populations able to differentiate *in vitro* has occurred *in vivo*. Indeed, these B cells have been likely primed by surface Ag—as expected—but also by core Ag—which means that at least some subsets of B cells in cultures have met their epitope processed by an APC *in vivo*. Ags other than ENV, i.e. GAG and POL are internal Ags and must be processed or exposed (cell/virus lysis) for subsequent presentation to the relevant Ag-reactive B cell. This hypothesis was supported by the blocking studies which aimed at interrupting intercellular cooperation. Our results do not exclude the possibility that specific Ab production can be affected by exogenous- or accessory-cell derived-stimuli such as cytokines [24] along with IL-2 and IL-10 are two cytokines which affect almost all the activation and differentiation steps of B lymphocytes especially their maturation (Banchereau and Rousset, 1992), and rescue germinal center-B cells from apoptosis (Levy and Brouet, 1994). The role of accessory- or B cells derived-IL-6 and -TNF-$\alpha$ was found to be critically important; we did not test for the addition of NAbs to IL-6 or TNF-$\alpha$ in individual cultures since it is well established by others and ourselves that this affects all Ab production *in vitro* in most individual culture conditions (Garraud *et al.*, 1996; Fournier *et al.*, 2002).

Of note, it is assumed that most Ag-reactive B cells recognize the native form of the envelope gp120/160 Ag, exposed by a cell-free or a cell-bound virus. We show here that patients' blood B lymphocytes in uncontrolled HIV infection (no-anti viral therapy) were capable of producing *in vitro* Abs against various HIV Ags unlike the majority of patients with controlled viremia. Our data is consistent with other published studies (Zamarchi *et al.*, 2002) since there is not *stricto sensu* a defined correlation between the initial VL and the capacity to produce Abs. We propose instead that this parameter parallels the length of exposure to the virus and/or the absence of therapeutic control.

We report here that HIV-infected individuals—even AIDS patients with disorganized lymph node machinery (Legendre *et al.*, 1998)—can produce Abs reactive to HIV envelope Ags which—at least in an *in vitro* model—bind their cognate Ags, and possibly also against Ags from concurrent infections (ongoing experiments). Patients seem thus retain the capacity of developing certain Ab responses. This property could be appropriately targeted by immuno-interventions against HIV but also apportunistic and/or concurrent infections.

### *Acknowledgements*

We are greatly indebted to the patients who accepted to be enrolled in the study, and to the nurses and medical staff members who contributed to the clinical aspect of this study, particularly to Ms J. Leal (Institut Pasteur, Bangui); Dr C. Defontaine, Dr A. Frésard, Ms A-M. Lantner (CHU, St-Etienne). We also deeply thank Dr T. Bourlet, Dr O. Delézay, Ms S. Peruchon, Ms F. Duplat, and Mr D. Laurent (Université de St-Etienne) for their kind help in managing data; Dr R. El Habib (Aventis-Pasteur, Marcy l'Etoile, France) and Dr F. Brière (Schering-Plough, Dardilly, France) for their kind gift of critical reagents. We also wish to express our gratitude to Pr J.-L. Durosoir, Pr Y. Buisson and Pr F. Barré-Sinoussi (Institut Pasteur, Paris, and Réseau International des Instituts Pasteur et Instituts Associés); and Dr A. Talarmin (Institut Pasteur, Bangui) for their invaluable support. This work has been supported by a grant number (2000/028) by the ANRS, the French National Agency for AIDS Research. The study has also been granted in part by the "Convention Interrégionale du Massif Central"-"Réseau switch"- MENRT 01Y0242b. L. Béniguel holds a fellowship from the French Ministry for Education, Research and Technology (MENRT).

### *References*

Amadori, A., De Rossi, A., Faulkner-Valle, G.P., *et al.* (1988) "Spontaneous *in vitro* production of virus-specific antibody by lymphocytes from HIV-infected subjects", *Clin. Immunol. Immunopathol.* **46**, 342–351.

Baba, T.W., Liska, V., Hofmann-Lehmann, R., *et al.* (2000) "Human neutralizing monoclonal antibodies of the IgG1 subtype protect against mucosal simian–human immunodeficiency virus infection", *Nat. Med.* **6**, 200–206.

Baize, S., Leroy, E.M., Georges-Courbot, M.C., *et al.* (1999) "Defective humoral responses and extensive intravascular apoptosis are associated with fatal outcome in Ebola virus-infected patients", *Nat. Med.* **5**, 423–426.

Banchereau, J. and Rousset, F. (1992) "Human B lymphocytes: phenotype, proliferation and differentiation", *Adv. Immunol.* **52**, 125–262.

Begaud, E., Feindirongai, G., Versmisse, P., *et al.* (2003) "Broad spectrum of coreceptor usage and rapid disease progression in HIV-1-infected individuals from Central African Republic", *AIDS Res. Hum. Retroviruses* **19**, 551–560.

Beniguel, L., Begaud, E., Cognasse, F., *et al.* (2004) "Identification of germinal center B cells in blood from HIV-infected drug naive individuals in Central Africa", *Clin. Dev. Immunol.* **11**, 23–27.

Bourlet, T., Cazorla, C., Berthelot, P., *et al.* (2001) "Compartmentalization of HIV-1 according to antiretroviral therapy: viral loads are correlated in blood and semen but poorly in blood and saliva", *Aids* **15**, 284–285.

Cognasse, F., Beniguel, L., El Habib, R., *et al.* (2003) "HIV-gp160 modulates differentially the production *in vitro* of IgG, IgA and cytokines by blood and tonsil B lymphocytes from HIV-negative individuals", *Clin. Exp. Immunol.* **132**, 304–318.

Defrance, T., Casamayor-Palleja, M. and Krammer, P.H. (2002) "The life and death of a B cell", *Adv. Cancer Res.* **86**, 195–225.

Devito, C., Hinkula, J., Kaul, R., *et al.* (2000) "Mucosal and plasma IgA from HIV-1-exposed uninfected individuals inhibit HIV-1 transcytosis across human epithelial cells", *Aids* **14**, 1917–1920.

Fauci, A.S., Pantaleo, G., Stanley, S., *et al.* (1996) "Immunopathogenic mechanisms of HIV infection", *Ann. Intern. Med.* **124**, 654–663.

Fournier, A.M., Fondere, J.M., Alix-Panabieres, C., *et al.* (2002) "Spontaneous secretion of immunoglobulins and anti-HIV-1 antibodies by *in vivo* activated B lymphocytes from HIV-1-infected subjects", *Clin. Immunol.* **103**, 98–109.

Garin, B., Glaziou, P., Kassa-Kelembho, E., *et al.* (1997) "High mortality rates among patients with tuberculosis in Bangui, Central African Republic", *Lancet* **350**, 1298.

Garraud, O., Nkenfou, C., Bradley, J.E., *et al.* (1995) "Identification of recombinant filarial proteins capable of inducing polyclonal and antigen-specific IgE and IgG4 antibodies", *J. Immunol.* **155**, 1316–1325.

Garraud, O., Nkenfou, C., Bradley, J.E., *et al.* (1996) "Differential regulation of antigen-specific IgG4 and IgE antibodies in response to recombinant filarial proteins", *Int. Immunol.* **8**, 1841–1848.

Garraud, O., Diouf, A., Nguer, C.M., *et al.* (2001) "Experimental IgG antibody production *in vitro* by peripheral blood and tonsil surface gamma+ B lymphocytes from *Plasmodium falciparum*-immune West Africans", *Scand. J. Immunol.* **54**, 606–612.

Germani, Y., Minssart, P., Vohito, M., *et al.* (1998) "Etiologies of acute, persistent, and dysenteric diarrheas in adults in Bangui, Central African Republic, in relation to HIV status", *Am. J. Trop. Med. Hyg.* **59**, 1008–1014.

Greenberg, A.E., Coulibaly, I.M., Kadio, A., *et al.* (1997) "Impact of the 1994 expanded World Health Organization AIDS case definition on AIDS surveillance in university hospitals and tuberculosis centers in Cote d'Ivoire", *Aids* **11**, 1867–1872.

Legendre, C., Raphael, M., Gras, G., *et al.* (1998) "CD80 expression is decreased in hyperplastic lymph nodes of HIV+ patients", *Int. Immunol.* **10**, 1847–1851.

Levy, Y. and Brouet, J.C. (1994) "Interleukin-10 prevents spontaneous death of germinal center B cells by induction of the bcl-2 protein", *J. Clin. Investig.* **93**, 424–428.

Lukwiya, M., Rizzardini, G., Trabattoni, D., *et al.* (2001) "Evaluation of immune activation in HIV-infected and uninfected African individuals by single-cell analysis of cytokine production", *J. Acquir. Immune Defic. Syndr.* **28**, 429–436.

Mascola, J.R., Lewis, M.G., Stiegler, G., *et al.* (1999) "Protection of Macaques against pathogenic simian/human immunodeficiency virus 89.6PD by passive transfer of neutralizing antibodies", *J. Virol.* **73**, 4009–4018.

Menard, D., Mandeng, M.J., Tothy, M.B., *et al.* (2003) "Immunohematological reference ranges for adults from the Central African Republic", *Clin. Diagn. Lab. Immunol.* **10**, 443–445.

Moja, P., Tranchat, C., Tchou, I., *et al.* (2000) "Neutralization of human immunodeficiency virus type 1 (HIV-1) mediated by parotid IgA of HIV-1-infected patients", *J. Infect. Dis.* **181**, 1607–1613.

Opravil, M., Ledergerber, B., Furrer, H., *et al.* (2002) "Clinical efficacy of early initiation of HAART in patients with asymptomatic HIV infection and CD4 cell count $> 350 \times 10(6)/l$", *Aids* **16**, 1371–1381.

Richard, Y., Lefevre, E., Krzysiek, R., *et al.* (2002) In: Cossarizza, A. and Kaplan, D., eds, Cellular Aspects of HIV Infection (Wiley-Liss, New York), pp 69–102.

Vendrell, J.P., Segondy, M., Ducos, J., *et al.* (1991) "Analysis of the spontaneous *in vitro* anti-HIV-1 antibody secretion by peripheral blood mononuclear cells in HIV-1 infection", *Clin. Exp. Immunol.* **83**, 197–202.

Zamarchi, R., Barelli, A., Borri, A., *et al.* (2002) "Cell activation in peripheral blood and lymph nodes during HIV infection", *Aids* **16**, 1217–1226.

